# Perspective of Key Healthcare Professionals on Antimicrobial Resistance and Stewardship Programs: A Multicenter Cross-Sectional Study From Pakistan

**DOI:** 10.3389/fphar.2019.01520

**Published:** 2020-01-10

**Authors:** Khezar Hayat, Meagen Rosenthal, Ali Hassan Gillani, Jie Chang, Wenjing Ji, Caijun Yang, Minghuan Jiang, Mingyue Zhao, Yu Fang

**Affiliations:** ^1^ Department of Pharmacy Administration and Clinical Pharmacy, School of Pharmacy, Xi’an Jiaotong University, Xi’an, China; ^2^ Center for Drug Safety and Policy Research, Xi’an Jiaotong University, Xi’an, China; ^3^ Shaanxi Center for Health Reform and Development Research, Xi’an, China; ^4^ Institute of Pharmaceutical Sciences, University of Veterinary and Animal Sciences, Lahore, Pakistan; ^5^ Department of Pharmacy Administration, School of Pharmacy, University of Mississippi, Mississippi, MS, United States

**Keywords:** antimicrobial resistance, antimicrobial stewardship programs, antibiotic resistance, healthcare professionals, Pakistan, physicians, nurses, pharmacists

## Abstract

**Background:** Antimicrobial resistance (AMR) is an increasing global threat, and hospital-based antimicrobial stewardship programs (ASPs) are one of the effective approaches to tackle AMR globally. This study was intended to determine the attitude of key healthcare professionals (HCPs), including physicians, nurses, and hospital pharmacists, towards AMR and hospital ASPs.

**Methods:** A cross-sectional study design was used to collect data from HCPs employed in public teaching hospitals of Punjab, Pakistan, from January 2019 to March 2019. A cluster-stratified sampling method was applied. Descriptive statistics, Mann Whitney and Kruskal Wallis tests were used for analysis.

**Results:** A response rate of 81.3% (881/1083) for the surveys was obtained. The majority of the physicians (247/410, 60.2%) perceived AMR to be a serious problem in Pakistani hospitals (*p* < 0.001). Most of the HCPs considered improving antimicrobial prescribing (580/881, 65.8%; *p* < 0.001) accompanied by the introduction of prospective audit with feedback (301/881, 75.8%; *p* < 0.001), formulary restriction (227/881, 57.2%; *p* = 0.004) and regular educational activities (300/881, 75.6%; *p* = 0.015) as effective ASP methods to implement hospital ASPs in Pakistan. A significant association was found between median AMR and ASP scores with age, years of experience, and types of HCPs (*p* < 0.05).

**Conclusions:** The attitude of most of the HCPs was observed to be positive towards hospital-based ASPs regardless of their poor awareness about ASPs. The important strategies, including prospective audit with feedback and regular educational sessions proposed by HCPs, will support the initiation and development of local ASPs for Pakistani hospitals.

## Introduction

Antimicrobial resistance (AMR) is regarded as one of the most daunting challenges that the world is currently facing (Septimus, 2018). It has strongly amplified the risk of mortality and morbidity.(Huttner et al., 2013) In fact, it is estimated that between 700,000 and several million people who suffer infectious diseases from resistant microbes die worldwide every year (O’Neill, 2016). Furthermore, in Europe, AMR has directly contributed to the deaths of 33,000 people per year (Cassini et al., 2019). AMR has also increased the cost of medical treatment every year. For instance, the cost of treating AMR was estimated to be 1.68 billion US$ (Founou et al., 2017; European Commission, 2017). Additionally, 3.5 billion US$ per year will be needed for the next ten years to treat antimicrobial-resistant infections as predicted by the Organization for Economic Co-operation and Development (OECD, 2018; Hofer, 2019).

The catastrophic impact of AMR in developing countries is higher in magnitude and intensity, possibly due to the increased prevalence of resistant infections than in developed countries (O’Neill, 2016). It was revealed in a report issued by the World Health Organization (WHO) that 45% of deaths in South-East Asia are attributed to resistant bacterial infections, and resistant *K. pneumonia* was found to be involved in 81% of the deaths (World Health Organization, 2014). Furthermore, several reports have also indicated higher presence of methicillin-resistant *S. aureus* in different healthcare settings of developing countries including Morocco (14.4%), Ivory Coast (16.8%), Kenya (27.7%), Nigeria (29.6%), Ethiopia (42.8%), South Africa (52%), and Cameroon (72%) (Kejela and Bacha, 2013; Njoungang et al., 2015; Perovic et al., 2015).

Pakistan, being a developing country, is also increasingly facing this problem (Centre for Disease Dynamics, Economics and Policy, 2018c; Khan et al., 2019). A recent study conducted in a tertiary hospital found all *Acinetobacter* and more than 70% of *P. aeruginosa* isolates to be multidrug-resistant (MDR). Moreover, the resistance against third-generation cephalosporins was also noted to be higher (i.e., 85% for ceftazidime, 84% for ceftriaxone, and 87% for cefotaxime). The emergence of resistance against colistin is further worsening the situation, which is why Pakistan recently launched its National Action Plan (NAP) to help reduce AMR (Khan et al., 2009; Nahid et al., 2013; Saleem et al., 2018; Centre for Disease Dynamics, Economics and Policy, 2018a).

In Pakistan, the unjustified use of antibiotics in both the hospital and community settings is widespread. Surprisingly, the overall consumption of antibiotics has increased by 65% from 2000 to 2010 (Klein et al., 2018). Several causes of the irrational use of antibiotics in Pakistan include: an inadequate number of trained medical staff such as infectious disease (ID) physicians and clinical pharmacists, a lack of standard diagnostic labs, the availability of antibiotics over the counter, patients’ self-medication, poor public awareness about the use of antibiotics, and the prescribing of antibiotics by unlicensed practitioners (Gillani et al., 2017; Nazir and Azim, 2017; Hussnain, 2018; Muhammad Majid Aziz et al., 2018; Centre for Disease Dynamics, Economics and Policy, 2018b; Hayat et al., 2019b). Moreover, a recent point prevalence survey conducted in a range of hospitals in Punjab, Pakistan, showed the inappropriate prescribing of antibiotics (Saleem et al., 2019b). There is also a high rate of healthcare-associated infections (HAIs) in hospitals in Pakistan that need to be addressed alongside improving antimicrobial prescribing (Saleem et al., 2019a).

A hospital based-antimicrobial stewardship program (ASP) is regarded as a globally accepted tool to address the challenge of AMR (Cunha, 2018). The focus of ASPs is to rationalize the use of antibiotics, to enhance clinical outcomes, to limit the growing pace of AMR, and to shrink the medical cost associated with managing resistant infections (Society for Healthcare Epidemiology of America, 2007). The key stakeholders of hospital ASPs are ID physicians, clinical pharmacists, and nurses (Van Parys et al., 2016). These programs have already been implemented in numerous countries in different hospital settings, demonstrating their merits on a patient’s quality of life, and reduction in AMR (Brink et al., 2016; Polinski et al., 2017; Brotherton, 2018).

In Pakistan, the efforts to develop regional hospital-based ASPs were started in 2014 by Medical Microbiology and Infectious Diseases Society (Medical Microbiology and Infectious Diseases Society of Pakistan, 2014). However, its implementation in hospitals is still a challenging task (Cox et al., 2017; Fadare et al., 2019). Healthcare professionals (HCPs) working in hospitals should have adequate knowledge and awareness of AMR, its triggering factors, and strategies required to cope with AMR (Labi et al., 2018; Nicholson et al., 2018; Hayat et al., 2019a; Kalungia et al., 2019). Different professionals will have different views which could affect the functioning of hospital ASPs (Venugopalan et al., 2016; Salsgiver et al., 2018). Furthermore, the success of implementing ASPs in hospitals is highly dependent on the joint efforts of HCPs, including ID physicians, pharmacists, and nurses. Therefore, the current study was designed to investigate the views of HCPs working in teaching hospitals towards AMR and hospital ASPs in teaching hospitals of Punjab, Pakistan.

## Materials and Methods

### Study Design and Settings

Pakistan is one of the renowned countries of Asia, comprised of four provinces, including Punjab, Khyber Pakhtunkhwa, Balochistan, and Sindh (Pakistan Bureau of Statistics, 2017). Among all provinces, Punjab is the most developed province and ranks first in terms of population and GDP. It is further divided into nine administrative divisions. This cross-sectional and multicenter study was conducted among six divisions of Punjab having distinct GDP and socio-economic levels.

### Study Instrument

A literature review was undertaken to construct and conceptualize the questionnaire (García et al., 2011; Cotta et al., 2014; Broom et al., 2016; Baur et al., 2017; Shahid et al., 2017; Nellums et al., 2018; Sutthiruk et al., 2018). An effort was made to make the questionnaire simple and brief. Moreover, a qualitative study conducted between January 2018 and March 2018 also allowed the further refinement of the questionnaire (Hayat et al., 2019b). Content and face validity of the first version of the questionnaire was established by an expert panel of the multidisciplinary research team. Field tests were also conducted on a small group of HCPs (data were not included). Changes were made after gaining the feedback of experts, as a few questions were hard to understand and were therefore removed or modified.

The final version of the questionnaire comprised of 34-items and had six sections. The demographic information about HCPs, including the type of profession, age, gender, experience, and specialty, was asked in the first section of the questionnaire. The second and third sections of the questionnaire contained four questions related to awareness of AMR and five questions related to the triggering factors of AMR rated on a 7-point Likert scale (1 = not a problem to 7 = very serious problem and 1 = does not contribute to 7 = strongly contribute). Ten Questions related to patient care and ways to eradicate AMR were asked in the fourth section of the questionnaire, with options 1 = strongly disagree to 7 = strongly agree. The fifth section of the questionnaire focused on ASP strategies comprised of five questions. Here again, a 7-point Likert scale was opted (1 = very unhelpful to 7 = very helpful). The last section of the questionnaire has four questions about the previous involvement of HCPs in AMR and ASPs with three options “yes,” “no,” and “do not know.”

A pilot study was conducted on thirty HCPs to ensure the internal consistency of the questionnaire (the data were not included in the final analysis). The value of Cronbach alpha measured for sections two to six was more than seven indicating an acceptable level of internal consistency.

### Sampling

**Figure 1:**
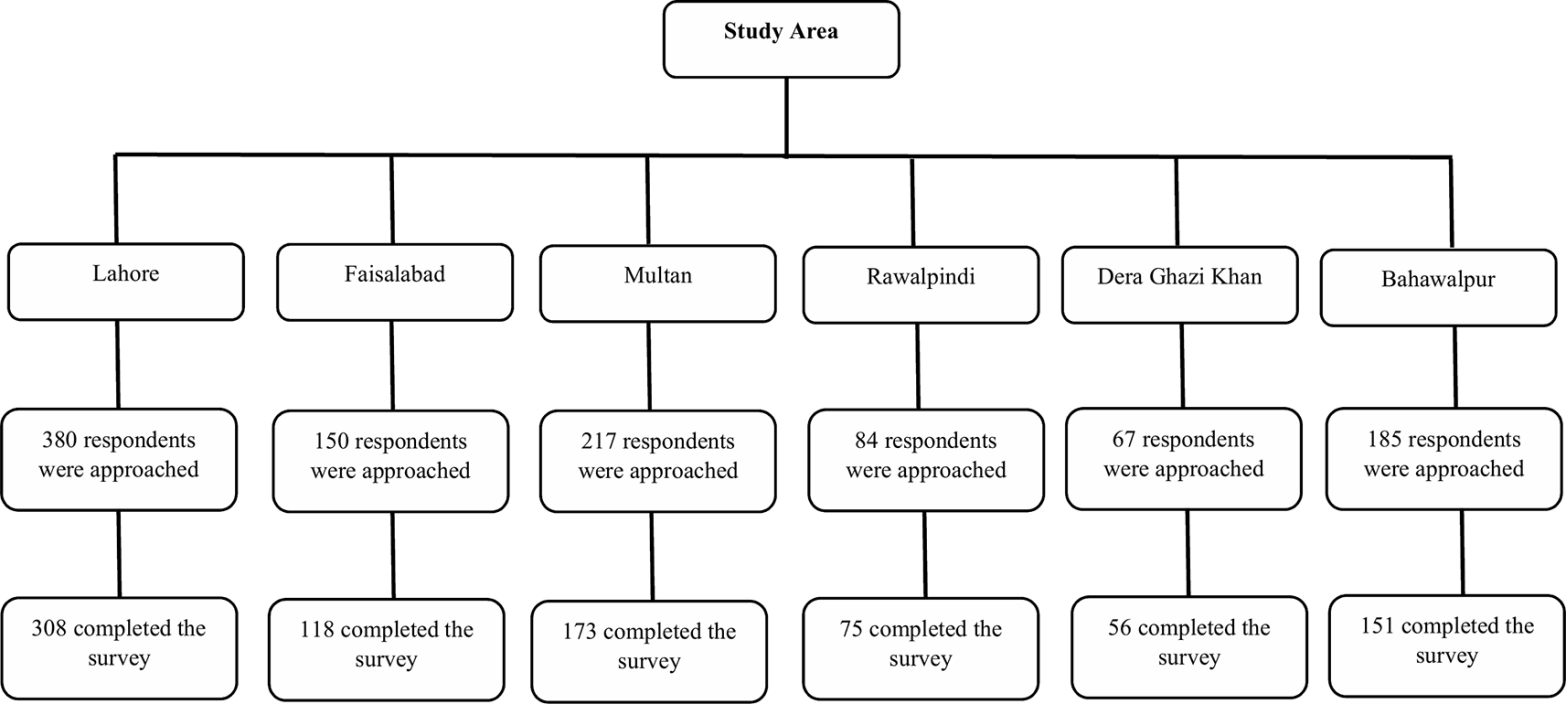
Number of healthcare professionals from each division of Punjab.

A multistage cluster-stratified sampling method was used to recruit HCPs, including physicians, nurses, and hospital pharmacists. In the first stage, six administrative divisions (Lahore, Faisalabad, Multan, Bahawalpur, Dera Ghazi Khan, and Rawalpindi) of the Punjab province were selected out of nine by cluster sampling, with names of divisions drawn from a hat (**Figure 1**). These divisions are distinct in terms of socioeconomic status and GDP. In the second stage of sampling, one public teaching hospital was selected randomly, with names of hospitals being drawn out of a hat for each chosen administrative division. Lastly, HCPs working in different departments such as medicine, surgery, operating rooms, ophthalmology, emergency, intensive care units, and obstetrics and gynecology were approached by stratified sampling method. Raosoft, an online sample size calculator, was used to determine the sample size with a 5% margin of errors, a 50% response rate, and a 95% confidence interval (Raosoft, 2015). The sample size for 100,375 registered physicians and 40,000 registered nurses was 383 and 381, respectively. However, all hospital pharmacists working in the surveyed hospital were recruited in this study. By considering a 30% non-response rate, we approached 498 physicians and 495 nurses. Only HCPs working in the public teaching hospital were included, whereas, HCPs working in non-teaching public hospitals or private hospitals were excluded.

### Data Collection

Trained data collectors with a pharmacy background collected the data from HCPs. The principal investigator (PI) informed all the data collectors about the aims and objectives of the study. To approach different HCPs, data collectors visited six teaching hospitals located in six administrative divisions of Punjab. The in-charge physician of the ward or department was briefed about the study purpose, and later facilitated the distribution of the questionnaire among HCPs. The data collectors addressed all the queries that arose from the HCPs. The PI also inspected the working of data collectors by conducting unscheduled and random visits to the hospitals to ensure the quality of the data collection. All the participating HCPs provided verbal consent before completing the questionnaire.

### Data Analysis

To summarize the data, and calculate frequencies, and percentages the Statistical Package for the Social Sciences (SPSS Inc, version 18, IBM, Chicago, IL, USA) was used. Kolmogorov–Smirnov and Shapiro–Wilk tests were employed to detect the data normality. Since the data did not follow the normal distribution, median, and interquartile ranges (IQRs) were measured. The categorical data were presented in proportions that agreed with a “6” and “7” Likert scale response. Mann-Whitney and Kruskal-Wallis tests were used on continuous data. Median AMR scores (measuring awareness about AMR and its contributing factors), AMR eradication scores (measuring AMR eradication approaches), and ASP scores (measuring ASP strategies) were also calculated, which were compared using demographic variables. A *p*-value <0.05 was set to be statistically significant. Post-hoc analysis (Bonferroni correction) was also carried out to determine the difference in intergroup variables. In order to prevent the inflation of type one error, the *p*-value was based on the total number of comparisons, such as for three group *p*-value < 0.017 was considered statistically significant (*p*-value = 0.05/3 = 0.017). Based on the results of the Kruskal–Wallis test, a follow-up analysis (the Mann–Whitney) was also conducted to determine where exactly the statistically significant difference occurs among the different HCPs (physicians, pharmacists, and nurses). Moreover, logistic regression was also applied to comparative estimates.

### Ethics Approval

The Biomedical Ethics Committee of Xi’an Jiaotong University approved the study (no. 2018-528). Additionally, permission to conduct this study was also taken from the University of Veterinary and Animal Sciences. Respondents were informed of the voluntary nature of their participation, and confidentiality was guaranteed.

## Results

### Demographic Information

There was a total of 881 HCPs completed the survey, of whom 46.5% (410) were physicians, 45.1% (397) nurses, and 8.4% (74) pharmacists. The overall response rate was 81.4% (physicians = 82.3%, nurses = 80.0%, and pharmacists = 83.1%). Many of the participating HCPs (537/881, 61.0%) were female as the nursing staff in Pakistan is mostly comprised of female nurses. Most of the HCPs (330/881, 37.5%) were aged between 25-30 years. A large proportion of HCPs were working in the medicine (180/881, 20.4%), pediatric (123/881, 14.1%), and surgery (115/881, 13.1%) departments. The majority of the HCPs had 1–5 years of clinical experience (**Table 1**).

**Table 1:** Demographic characteristics of healthcare professionals (n = 881).

Variable	Frequency (n)	Percentage (%)
Gender		
Male	344	39.0
Female	537	61.0
Types of healthcare professionals		
Physician	410	46.5
Pharmacist	74	8.4
Nurse	397	45.1
Age (years)		
<25	74	8.4
25 to 30	330	37.5
31 to 35	268	30.4
36 to 40	158	17.9
>40	51	5.8
Experience (years)		
<1	19	2.2
1 to 5	363	41.2
6 to 10	242	27.5
11 to 20	173	19.6
>20	84	9.5
Hospital departments		
Emergency	59	6.7
Intensive care units*	138	15.7
Medicine	180	20.4
Obstetrics & gynecology	102	11.6
Operating room	24	2.7
Ophthalmology	66	7.5
Pharmacy	74	8.4
Pediatrics	123	14.0
Surgery	115	13.1
Bed capacity of hospitals (n = 6)		
<1,000	3	50
1,000 to 1,500	1	16.7
>1,500	2	33.3

*Intensive care units of medicine, surgery, pediatrics, and obstetrics and gynecology are included.

### Views About AMR

More physicians (247/410, 60.2%) than pharmacists (39/74, 52.7%) and nurses (197/397, 49.6%) viewed AMR to be a serious problem in Pakistani hospitals (*p* < 0.001). However, physicians (212/410, 51.7%) were less likely to consider AMR as a serious threat in their practicing hospitals compared to pharmacists (54/74, 72.9%) and nurses (217/397, 54.7%). The greatest number of HCPs (514/881, 58.3%) deemed AMR as a serious issue in the Pakistani community outside of the hospital (*p* < 0.001) (**Table 2**).

**Table 2:** Perception of healthcare professionals towards antimicrobial resistance in agreement’ (i.e., with a “6” and “7” Likert scale response) n (%).

	Physicians (n = 410)	Pharmacists (n = 74)	Nurses (n = 397)	Total (n = 881)	Median (IQR)	*p-*value*
**Indicate how serious a problem you believe antimicrobial resistance is in the following places†**						
Worldwide	233 (57.7)^	43 (58.1)	94 (23.7)^#^	370 (41.9)	6(2)	<0.001
Pakistani hospitals	247 (60.2)^	39 (52.7)	197 (49.6)^#^	483 (54.8)	6(2)	<0.001
Pakistani community	262 (63.9)^	63 (85.1)^	189 (47.6)^#^	514 (58.3)	6(1)	<0.001
Your hospitals	212 (51.7)^	54 (72.9)^	217 (54.7)^#^	483 (54.8)	6(2)	0.001
**Indicate how strongly you believe the following contribute to antimicrobial resistance in Pakistan‡**						
Use of antimicrobials in Pakistani animals/agricultural sectors	184 (44.9)^	21 (28.4)^	195 (49.1)^#^	400 (45.4)	5(2)	<0.001
Use of antimicrobials in the Pakistani community	268 (65.4)	40 (54.1)	240 (60.5)	548 (62.2)	6(2)	0.033
Use of antimicrobials in Pakistani hospitals	284 (69.3)	37 (50.0)^	267 (67.3)^#^	588 (66.7)	6(2)	0.001
Use of antimicrobials in my hospital	147 (35.9)^	30 (40.5)	219 (55.2)^#^	396 (44.9)	5(2)	0.008
Patient pressure for antibiotics as part of treatment	207 (50.5)	32 (43.2)	189 (47.6)	428 (48.6)	5(3)	0.433
Patients are able to buy antibiotics without a prescription from a physician	244 (59.5)^	39 (52.7)	189 (47.6)^#^	472 (53.6)	6(2)	<0.001

*Kruskal–Wallis test.

†Rated on a Likert scale from 1 (not a problem) to 7 (very serious problem).

‡Rated on a Likert scale from 1 (does not contribute) to 7 (strongly contributes).

^#^and ^shows significant difference at *p* < 0.017.

A large number of HCPs perceived that use of antimicrobials in the Pakistani community (n = 548/881, 62.2%) and hospitals (588/881, 66.7%) as being a triggering factor for AMR however, they were in less agreement (396/881, 44.9%) that use of antimicrobial in their hospitals was a contributing factor (p < 0.008). Most of the HCPs (400/881, 45.4%) did not agree that the use of antimicrobials in Pakistani animals and the agriculture sector lead to AMR (*p* < 0.001).

A majority of the physicians (244/410, 59.5%) compared to nurses (189/397, 47.6%) and pharmacists (39/74, 52.7%) agreed that use of antibiotics without a prescription is an important factor contributing to AMR (*p* < 0.001) (**Table 2**).

### Views About Patient Care and Eradication of AMR

Only a handful of the HCPs (325/881, 36.9%) deemed that AMR affects patients under their supervision (*p* = 0.014); however, a large number of HCPs (573/881, 65.0%) agreed that better use of antibiotics would reduce the momentum of resistant infections (*p* = 0.034). Similarly, 67.9% (599/881) HCPs believed that the irrational use of antibiotics is not ethical (*p* < 0.012).

Physicians (297/410, 72.4%) were more likely than pharmacists (51/74, 68.9%) and nurses (n = 232/397, 58.4%) to agree that improving prescribing of antimicrobials could decrease AMR (*p* < 0.001). More than 50% of the HCPs agreed that a formal policy for the use of antimicrobial (566/881, 64.2%) accompanied by local antimicrobial guidelines and protocols (n = 569/881, 64.6%) should be introduced in their hospitals (**Table 3**).

**Table 3:** Perception of healthcare professionals about patient care and strategies to eradicate antimicrobial resistance in agreement’ (i.e., with a “6” and “7” Likert scale response) n (%).

Questions‡	Physicians (n = 410)	Pharmacists (n = 74)	Nurses (n = 397)	Total (n = 881)	Median (IQR)	*p-*value*
Antimicrobial resistance affects patients under my care.	168 (40.9)^	20 (27.0)	137 (34.5)^#^	325 (36.9)	5(2)	0.014
Better use of antibiotics will reduce problems with antibiotic-resistant organisms	267 (65.1)	39 (52.7)	267 (67.3)	573 (65.0)	6(2)	0.034
Inappropriate use of antibiotics can harm patients	268 (65.4)	46 (62.2)	271 (68.3)	585 (66.4)	6(2)	0.111
Inappropriate use of antibiotics is professionally unethical.	275 (67.1)	43 (58.1)^	281 (70.8)^#^	599 (67.9)	6(2)	0.012
Improving antimicrobial prescribing at your hospital will help decrease antimicrobial resistance at the hospital.	297 (72.4)^	51 (68.9)	232 (58.4)^#^	580 (65.8)	6(2)	<0.001
A formal policy for the use of antimicrobials should be introduced in my hospital.	233 (56.8)	49 (66.2)	284 (71.5)	566 (64.2)	6(2)	0.021
Local antimicrobial guidelines and protocols should be introduced in my hospital.	266 (64.9)	41 (55.4)	262 (65.9)	569 (64.6)	6(2)	0.379
A computer application which gives advice on the selection and duration of antimicrobial therapy for patients should be introduced in my hospital.	263 (64.1)^	32 (43.2)^#^	147 (37.0)^#^	442 (50.2)	6(2)	<0.001
A team consisting of a Specialist Physician and Pharmacist, providing individualized antimicrobial prescribing advice and feedback should be introduced in my hospital.	214 (52.2)^	48 (64.9)	282 (71.0)^#^	544 (61.7)	6(2)	0.002
I would be willing to participate in any initiatives involving antimicrobial use in my hospital.	207 (50.5)^	37 (50)	264 (66.5)^#^	508 (57.7)	6(2)	0.004

*Kruskal–Wallis test.

‡Rated on a Likert scale from 1 (strongly disagree) to 7 (strongly agree).

^#^and ^shows significant difference at *p* < 0.017.

Nurses (147/397, 37.0%) were less in support than physicians (263/410, 64.1%) and pharmacists (32/74, 43.2%) of introducing a computer application to rationalize the use of antimicrobials (*p* < 0.001). Nevertheless, nurses (282/397, 71.0%) were more in agreement to establish an ASP team. Furthermore, most of the HCPs (508/881, 57.7%) showed a positive attitude to become part of an ASP team (*p* < 0.004).

### Attitude Towards ASP Strategies

More of the nurses (301/397, 75.8%) than physicians (274/410, 66.8%) and pharmacists (42/74, 56.8%) considered regular hospital-wide audit and feedback on antibiotics as being a helpful ASP strategy (*p* < 0.001). Many HCPs (629/881, 71.4%) were not in favor of restricting the prescribing of all antibiotics (*p* = 0.001); however, they (541/881, 61.4%) showed a positive attitude to limit the prescribing of certain antibiotics (*p* = 0.004). The other imperative approaches to which HCPs agreed were readily accessible microbiological data (581/881, 65.9%) and regular education sessions (672/881, 76.3%) (**Table 4**).

**Table 4:** Attitude of healthcare professionals towards strategies of hospital ASPs in agreement’ (i.e., with a “6” and “7” Likert scale response) n (%).

Questions‡	Physicians (n = 410)	Pharmacists (n = 74)	Nurses (n = 397)	Total (n = 881)	Median (IQR)	*p*-value*
Regular hospital-wide audit and feedback on antibiotic utilization	274 (66.8)^	42 (56.8)^	301 (75.8)^#^	617 (70.0)	6(2)	<0.001
Restriction of prescription of all antibiotics	147 (35.9)^	21 (28.4)	84 (21.2)^#^	252 (28.6)	3(4)	0.001
Restriction of prescription of certain antibiotics	271 (66.1)^	43 (58.1)^#^	227 (57.2)	541 (61.4)	6(2)	0.004
Readily accessible microbiological data and advice	297 (72.4)	49 (66.2)	235 (59.2)	581 (65.9)	6(2)	0.099
Regular educational sessions	318 (77.6)	54 (72.9)^	300 (75.6)^#^	672 (76.3)	7(1)	0.015

*Kruskal–Wallis test.

‡Rated on a Likert scale from 1 (strongly disagree) to 7 (strongly agree).

^#^and ^shows significant difference at *p* < 0.017.

### Previous Involvement in AMR and Hospital ASPs

**Figure 2:**
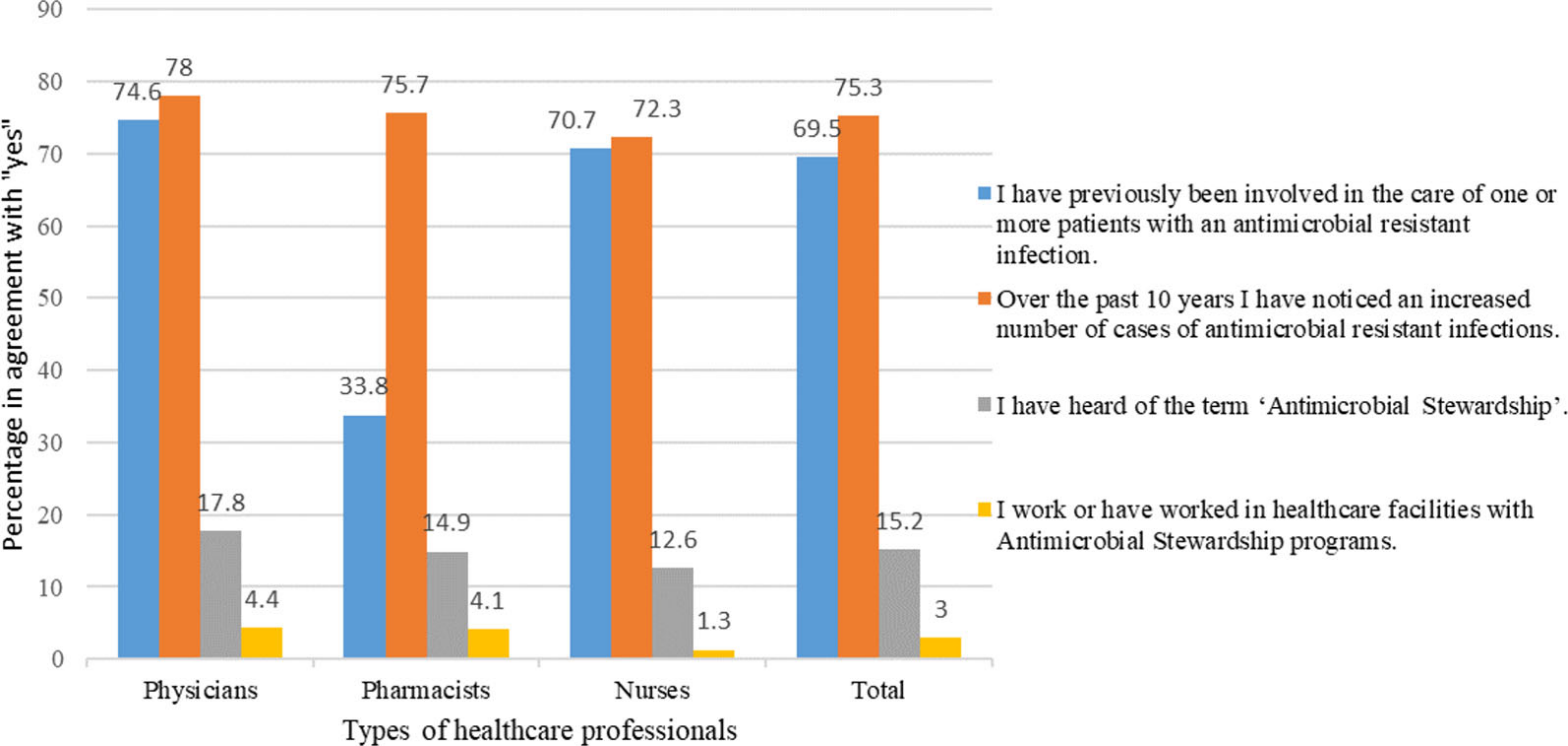
Previous engagement of healthcare professionals in AMR and ASPs.

A larger number of physicians (306/410, 74.6%) and nurses (281/397, 70.7%) than pharmacists (25/74, 33.8%) were involved in taking care of patients with resistant infections nevertheless, more than half of the HCPs (663/881, 75.3%) agreed that the rate of resistance is escalating over the last 10 years. Only a fraction of HCPs (134/881, 15.2%) had heard of an “antimicrobial stewardship program.” Moreover, only 3% (26/881) HCPs said that they have worked in a healthcare setting with a functional ASP (**Figure 2**).

### Association of Median Scores With Demographics

A significant association of types of HCPs was found with median attitude scores on AMR and ASP. The physicians were more positive in attitude than nurses towards AMR (Median scores: 6.0 vs. 5.5, *p* < 0.05). The median ASP attitude score of nurses was significantly higher than pharmacists (Median scores: 6.0 vs. 5.0, *p* < 0.05).

The age of the HCPs also had a significant association with median scores as HCPs between the ages of 36–40 years had more awareness than younger HCPs about AMR (Median scores: 6.0 vs 5.5, *p* < 0.05) and ASP strategies (Median scores: 7.0 vs 6.0, *p* < 0.05).

The experience of HCPs also significantly affected the median AMR and ASP scores. The HCPs with 6–10 years of experience had a more positive perception towards AMR than those HCPs with 1–5 years (Median scores: 6.0 vs. 5.0, *p* < 0.05) (**Table 5**).

**Table 5:** Median scores of healthcare professionals with IQR about their perception towards AMR, AMR eradication, and ASP methods.

Variables	AMR score median (IQR)	*p-*value	AMR eradication score median (IQR)	*p*-value	ASP score median (IQR)	*p-*value
Gender*						
Male	5.5(1.50)	0.143	6.0(2.00)	0.920	6.0(2.00)	0.597
Female	5.5(1.50)		6.0(2.00)		6.0(2.00)	
Healthcare professionals**						
Physician	6.0(1.50)	0.016	6.0(2.00)	0.366	6.0(2.00)	
Pharmacist	5.5.(2.50)		6.0(2.00)		6.0(2.00)	
Nurses	5.5(1.00)		6.0(2.00)		7.0(2.00)	0.021
Age (years)**						
<25	5.0(4.00)		5.5(1.63)		6.0(2.00)	
25 to 30	5.5(1.13)		6.0(2.00)		6.0(2.00)	
31 to 35	6.0(1.50)		6.0(2.00)		6.0(2.00)	
36 to 40	6.0(2.00)	0.001	6.0(2.00)	0.039	7.0(2.00)	0.009
>40 years	6.0(5.50)		6.5(2.50)		7.0(3.00)	
Experience (years)**						
<1	5.0(1.00)		6.5(2.0)		5.0(3.00)	
1 to 5	5.0(1.00)		6.0(2.0)		6.0(2.0)	
6 to 10	6.0(2.000)	<0.001	6.0(2.0)	0.133	7.0(2.0)	0.006
11 to 20	6.0(2.00)		6.0(2.0)		7.0(2.0)	
>20	5.5(1.38)		6.0(2.0)		6.0(2.0)	

*Mann–Whitney Test.

**Kruskal–Wallis Test.

The logistic regression analysis showed that HCPs with experience 1–5 years [(odds ratio) OR: 3.824; 95% CI (1.351–10.826);*p* = 0.012] and physicians [(odds ratio) OR: 4.748; 95% CI (2.800–8.051);*p* < 0.001] were significantly more likely to involve in dealing patients with resistant infections than other HCPs (see **Supplementary File**).

## Discussion

This multicenter and multidisciplinary study provides the latest insight about the perspective of all key HCPs, including physicians, pharmacists, and nurses from a low- and middle-income country (LMIC).

The majority of each of the HCP respondents were of the view that AMR is a global threat, and it is a severe issue of Pakistani hospitals, including their practicing hospital. It has already reported in several studies that AMR is a global issue (Wood et al., 2012; Cotta et al., 2014; Sutthiruk et al., 2018). A recent single-site survey has also described that physicians consider AMR as a severe threat in a Pakistani hospital (Shahid et al., 2017). However, in our study, physicians (247/410, 60.2%) perceived AMR as a more serious issue in hospitals other than their own (212/410, 51.7%). Furthermore, only 35.9% (147/410) physicians believed that the use of antimicrobials in their hospitals contributes to AMR. This difference in views towards AMR problem will affect the development of local guidelines to treat resistant infections. Nevertheless, a lot of studies published have also found this inconsistent perception as physicians do not always believe that AMR affects their practice in their practicing hospitals (García et al., 2011; Navarro-San Francisco et al., 2013; Hayat et al., 2019c). This may be because the use of antibiotics without a prescription is common in some countries like Pakistan, China, Saudi Arabia, and Ethiopia and HCPs link the rise in AMR in hospitals to the irrational use of antibiotics in the community outside of the hospital (Abdulhak et al., 2011; García et al., 2011; Chang et al., 2017; Erku and Aberra, 2018; Hayat et al., 2019b).

A significant difference in perception about AMR was observed between HCPs as nurses were less likely to consider AMR as a serious problem in the community possibly due to their diminished role in community healthcare settings as they are mostly engaged in hospital settings, as also highlighted by a Thai study (Sutthiruk et al., 2018). Moreover, a study conducted in a hospital in Australia also concluded that only 38% of the nurses perceived AMR as a severe issue of the community (Cotta et al., 2014).

Improper and excessive use of antimicrobials in agriculture and animal sector is widespread across the world (Chang et al., 2015), but in our study, only 45.4% (400/881) HCPs believed that the use of antimicrobials affects AMR rate. Furthermore, pharmacists were the least likely to agree that agricultural use of antimicrobials impacted AMR. This lack of knowledge among different HCPs was also observed in a study conducted in Ghana may be due to their insufficient training and educational programs (Labi et al., 2018).

More than 50% of HCPs agreed that patients could buy antibiotics over the counter without any valid prescription (472/881, 53.6%) and this could contribute to AMR. These results comply with studies conducted in other developing countries, including China, Ethiopia, and Thailand (Chang et al., 2017; Erku and Aberra, 2018; Sutthiruk et al., 2018). Nurses were least aware of this practice because they worked only in a hospital. Moreover, they have no direct role in making treatment decisions, as their role is mainly focused on the administration and management of antibiotics in hospitals of Pakistan.

In accordance with the previous studies (Wood et al., 2012; Navarro-San Francisco et al., 2013; Cotta et al., 2014; Sutthiruk et al., 2018), only 36.9% (325/881) HCPs agreed with the statement that “AMR affects patients under their care” even though AMR is increasing day by day (Laxminarayan et al., 2013; Nellums et al., 2018). Furthermore, a handful of pharmacists were in agreement with this statement, as in Pakistan, hospital pharmacists are primarily involved in the provision of traditional services including dispensing of drugs and record-keeping and do not provide many clinical services (Azhar et al., 2009; Azhar et al., 2011).

More than half of the HCPs considered all AMR eradication strategies, such as policies and systems to improve antimicrobial prescribing accompanied by regional antimicrobial guidelines and protocols, helpful to limit the progression of AMR in Pakistan. The systems comprised of optimal antibiotic use have already shown effectiveness in terms of clinical benefits (Cotta et al., 2014; Cresswell et al., 2017). Therefore, the Society for Healthcare Epidemiology of America (SHEA) and Infectious Disease Society of America (IDSA) have recommended the implementation of such types of systems, including hospital-based ASPs in hospitals (Dellit et al., 2007; Barlam et al., 2016). In line with IDSA and SHEA, the CDC and the World Health Assembly have also focused on the rational use of antibiotics with the implementation of ASPs in all healthcare settings (Fishman et al., 2012; Mendelson and Matsoso, 2015). Many countries around the globe have already adopted these systems. For example, in Australia, hospital accreditation can only be granted if the hospital has implemented an ASP (Margaret Duguid and Cruickshank, 2011). Moreover, the Joint Commission of the US has also endorsed and mandated the implementation of hospital ASPs comprised of an ASP team, including ID physician and pharmacist(s) in all hospital settings (Joint Commission Perspectives, 2016). However, the developing countries like Pakistan are still struggling to implement hospital-based ASPs successfully due to the lack of resources, untrained medical professionals, and inadequate diagnostic facilities (Hayat et al., 2019b).

Formulary restriction and hospital-wide audits with feedback are two imperative hospital ASP strategies (Tavares et al., 2018). All participating HCP, including physicians, pharmacists, and nurses, considered educational programs, restriction of certain antibiotics, hospital audit and feedback, and readily accessible microbiological reports as useful approaches to implement ASPs in Pakistani hospitals. The effectiveness of these strategies is already evident in different countries to slow down AMR (Lai et al., 2016; Brotherton, 2018; Zhou and Ma, 2019).

In our study, many HCPs (612/881, 69.5%) were involved in taking care of patients with resistant infections, which further strengthens their perception of AMR as of grave concern. However, this is not always true as some studies have shown an inconsistent relationship in physicians who have taken care of patients with resistant infections, and the seriousness with which they take the AMR problem in their hospital (Cotta et al., 2014). For example, an Australian study indicated that 84% of the HCPs were treating patients with resistant microbial infections, but only 45% considered AMR as a severe issue (Cotta et al., 2014).

A poor understanding by all HCPs of ASP was found, as only 15.2% of HCPs were familiar with this term. This may be because hospital-based ASPs have not been implemented in all Pakistani hospitals, and HCPs lack awareness of and training towards this type of program. Furthermore, the lack of conferences and workshops about ASPs could be another reason. Training with short-term courses outlining core elements of ASPs should be offered to all HCPs prior to implement ASPs in Pakistan successfully (Silverberg et al., 2017). This point has also elaborated previously (Hayat et al., 2019b). Importantly, the vast majority of HCPs (508/881, 57.7%), including physicians, pharmacists, and nurses, were willing to play their prescribed roles after the implementation of hospital ASPs in Pakistan despite their currently inadequate ASP knowledge.

## Limitations

There are some limitations of this study that should be noted before policymaking based on our findings. First, this study was conducted in six of the nine administrative divisions of Punjab province. While more than half of the Pakistani population lives in Punjab, and the six divisions were selected by cluster sampling to minimize the bias, the findings may not apply to other parts of Pakistan. Second, the nature of participation of HCPs was voluntary, which could cause selection bias, and views of non-participating HCPs are unknown. Third, this study was carried out in tertiary care public hospitals of Punjab, and the attitudes of HCPs working in the non-public hospitals may differ. However, HCPs working in the public sector hospitals in Pakistan also work in the private sector during off-hours. Irrespective of the above limitations, this multidisciplinary and multicenter study highlights the latest views of all major stakeholders of ASPs towards AMR and ASPs from an LMIC. Furthermore, the Government of Pakistan may consider the findings of this study for the development of local guidelines to implement hospital ASPs across all healthcare settings.

## Conclusions

AMR was viewed as a serious issue by most of the HCPs. Formulary restriction and hospital-wide audit with feedback on antibiotic use accompanied with regular educational sessions are key strategies proposed by HCPs to implement hospital ASPs in Pakistan. The significant differences in perceptions and attitudes of different professionals towards AMR and ASPs found in this study will help to develop and tailor regional ASPs. Regardless of inadequate ASP familiarity, the attitude of most of the HCPs was positive about hospital-based ASPs.

## Data Availability Statement 

The datasets generated and/or analyzed during the current study are not publicly available due to privacy restrictions but are available from the corresponding author on reasonable request.

## Ethics Statement

The Medical Ethics Committee of Xi’an Jiaotong University (2018-528) approved the study. Additionally, permission to conduct this study was granted by the University of Veterinary and Animal Sciences. Respondents were informed of the voluntary nature of their participation, and confidentiality was guaranteed. Written informed consent for participation was not required for this study in accordance with the national legislation and the institutional requirements.

## Author Contributions

Conceptualization: KH and YF. Data curation: KH, AG, and MJ. Formal analysis: KH and JC. Investigation: KH. Methodology: KH and YF. Resources: KH and YF. Software: WJ, MZ and CY. Supervision: YF and MR. Writing—original draft: KH. Writing—review and editing: YF and MR. All authors read and approved the final manuscript.

## Funding

This work was funded by the National Natural Science Fund (71974156), “Young Talent Support Plan,” “High Achiever Plan” of the Health Science Center, Xi’an Jiaotong University, and the Central University Basic Research Fund (2015qngz05).

## Conflict of Interest

The authors declare that the research was conducted in the absence of any commercial or financial relationships that could be construed as a potential conflict of interest.
