# Supplementary material for: Perspective of Key Healthcare Professionals on Antimicrobial Resistance and Stewardship Programs: A Multicenter Cross-Sectional Study From Pakistan
Source: Front Pharmacol. 2020 Jan 10;10:1520. doi: 10.3389/fphar.2019.01520 (PMC6967405; doi:10.3389/fphar.2019.01520)
Supplement: Supplementary file 1 [file DataSheet_1.docx]

**Perspective of key healthcare professionals on antimicrobial resistance and stewardship programs: a multicenter cross-sectional study from Pakistan**

**(Part 1) Demographic information**

**1. Profession:**  Physician  Pharmacist  Nurse

**2. Medical or Surgical Specialty/working area: _________________________**

**3. Gender:**  Male  Female

**4. Age in Years:**

< 25 year n 25 to 30 years  31 to 35 years n 36 to 40 years  > 40 years

**5. Years of experience (post primary qualification eg; post MBBS, B Pharmacy/Pharm D, Bachelor in Nursing):**

< 1 year n1 to 5 years n 6 to 10 years n 11 to 20 years  > 20 years

**(Part 2) Indicate how serious a problem you believe antimicrobial resistance is in the following places (scale of 1=not a problem to 7=very serious problem).**

**6. World­wide**

1(Not a 2 n 3 n 4  5 6 7 (Very (problem) (serious problem)

**7. Pakistani Community**

1(Not a 2 n 3 n 4  5 6 7 (Very (problem) (serious problem)

**8. Pakistani Hospitals**

1(Not a 2 n 3 n 4  5 6 7 (Very (problem) (serious problem)

**9. Your hospital**

1(Not a 2 n 3 n 4  5 6 7 (Very (problem) (serious problem)

**(Part 3) Indicate how strongly you believe the following contribute to antimicrobial resistance in Pakistan (scale of 1=does not contribute to 7=strongly contribute).**

**10. Use of antimicrobials in Pakistani animals/agricultural sectors**

1(Does not 2 n 3 n 4  5 6 7 contribute) (Strongly contributes)

**11. Use of antimicrobials in the Pakistani community**

1(Does not 2 n 3 n 4  5 6 7 contribute) (Strongly contributes)

**12. Use of antimicrobials in Pakistani hospitals**

1(Does not 2 n 3 n 4  5 6 7 contribute) (Strongly contributes)

**13. Use of antimicrobials in my hospital**

1(Does not 2 n 3 n 4  5 6 7 contribute) (Strongly contributes)

**14. Patient pressure for antibiotics as part of treatment**

1(Does not 2 n 3 n 4  5 6 7 contribute) (Strongly contributes)

**15. Patients are able to buy antibiotics without a prescription from a physician**

1(Does not 2 n 3 n 4  5 6 7 contribute) (Strongly contributes)

**(Part 4) Indicate how strongly do you agree or disagree with the statements below (scale of 1=strongly disagree to 7=strongly agree).**

**16. Antimicrobial resistance affects patients under my care at my hospital.**

1(Strongly 2 n 3 n 4  5 6 7 disagree) (Strongly agree)

**17. Better use of antibiotics will reduce problems with antibiotic-resistant organisms.**

1(Strongly 2 n 3 n 4  5 6 7 disagree) (Strongly agree)

**18. Inappropriate use of antibiotics can harm patients.**

1(Strongly 2 n 3 n 4  5 6 7 disagree) (Strongly agree)

**19. Inappropriate use of antibiotics is professionally unethical.**

1(Strongly 2 n 3 n 4  5 6 7 disagree) (Strongly agree)

**20. Improving antimicrobial prescribing at your hospital will help decrease antimicrobial resistance at the hospital.**

1(Strongly 2 n 3 n 4  5 6 7 disagree) (Strongly agree)

**21. A formal policy for the use of antimicrobials should be introduced in my hospital.**

1(Strongly 2 n 3 n 4  5 6 7 disagree) (Strongly agree)

**22. Local antimicrobial guidelines and protocols should be introduced in my hospital.**

1(Strongly 2 n 3 n 4  5 6 7 disagree) (Strongly agree)

**23. A computer application which gives advice on selection and duration of antimicrobial therapy for patients should be introduced in my hospital.**

1(Strongly 2 n 3 n 4  5 6 7 disagree) (Strongly agree)

**24. A team consisting of a Specialist Physician and Pharmacist providing individualized antimicrobial prescribing advice and feedback should be introduced in my hospital.**

1(Strongly 2 n 3 n 4  5 6 7 disagree) (Strongly agree)

**25. I would be willing to participate in any initiatives involving antimicrobial use in my hospital.**

1(Strongly 2 n 3 n 4  5 6 7 disagree) (Strongly agree)

**(Part 5) Indicate what ASP measures do you think would be the most helpful in improving antibiotic utilization in Pakistan (scale of 1=very unhelpful) to 7=very helpful)).**

**26. Regular hospital-wide audit and feedback on antibiotic utilization**

1(very 2 n 3 n 4  5 6 7 (very unhelpful) helpful)

**27. Restriction of prescription of all antibiotics**

1(very 2 n 3 n 4  5 6 7 (very unhelpful) helpful)

**28. Restriction of prescription of certain antibiotics**

1(very 2 n 3 n 4  5 6 7 (very unhelpful) helpful)

**29. Readily accessible microbiological data and advice**

1(very 2 n 3 n 4  5 6 7 (very unhelpful) helpful)

**30. Regular educational sessions**

1(very 2 n 3 n 4  5 6 7 (very unhelpful) helpful)

**(Part 6) Previous involvement in AMR and hospitals ASPs.**

**31. I have previously been involved in the care of one or more patients with an antimicrobial resistant infection.**

1(Yes 2 (No) n 3 (Unsure)

**32. Over the past 10 years I have noticed an increased number of cases of antimicrobial resistant infections.**

1(Yes) 2 (No) n 3 (Unsure)

**33. I have heard of the term ‘Antimicrobial Stewardship’.**

1(Yes) 2 (No) n 3 (Unsure)

**34. I work or have worked in healthcare facilities with Antimicrobial Stewardship programs.**

1(Yes) 2 (No) n 3 (Unsure)

**Thank You for Completing the Survey**
