# Supplementary material for: Perspective of Key Healthcare Professionals on Antimicrobial Resistance and Stewardship Programs: A Multicenter Cross-Sectional Study From Pakistan
Source: Front Pharmacol. 2020 Jan 10;10:1520. doi: 10.3389/fphar.2019.01520 (PMC6967405; doi:10.3389/fphar.2019.01520)
Supplement: Supplementary file 2 [file Table_1.docx]

**Table: Association of socio-demographic characteristics and involvement in patients with resistant infections**

| **Variable** | **Involved in AMR patients (n=612) n (%)** | **Odds ratio (95% CI)** | **p-value** |
| --- | --- | --- | --- |
| Gender |  |  |  |
| Male | 243 (39.7) | 0.913 (0.680-1.226) | 0.545 |
| Female | 369 (60.3) | 1.000 | ------ |
| Types of healthcare professionals |  |  |  |
| Physician | 306 (50.0) | 4.748 (2.800- 8.051) | <0.001 |
| Pharmacist | 25 (4.1) | 0.823 (0.604- 1.123) | 0.219 |
| Nurse | 281 (45.9) | 1.000 | ------ |
| Age (years) |  |  |  |
| < 25 | 37 (6.0) | 2.127 (0.988-4.581) | 0.054 |
| 25 to 30 | 235 (38.4) | 1.068 (0.552-2.066) | 0.844 |
| 31 to 35 | 189 (30.9) | 1.105 (0.566-2.156) | 0.770 |
| 36 to 40 | 110 (18.0) | 1.153 (0.571-2.328) | 0.691 |
| > 40 | 41 (6.7) | 1.000 | ------ |
| Experience (years) |  |  |  |
| < 1 | 7 (1.1) | 0.983 (0.588-1.642) | 0.947 |
| 1 to 5 | 252 (41.2) | 3.824 (1.351-10.826) | 0.012 |
| 6 to 10 | 173 (28.3) | 0.890 (518-1.527) | 0.672 |
| 11 to 20 | 122 (19.9) | 0.933 (0.529-1.643) | 0.809 |
| > 20 | 58 (9.5) | 1.000 | ------ |
